# Eyes Open on Sleep and Wake: In Vivo to In Silico Neural Networks

**DOI:** 10.1155/2016/1478684

**Published:** 2016-01-14

**Authors:** Amaury Vanvinckenroye, Gilles Vandewalle, Christophe Phillips, Sarah L. Chellappa

**Affiliations:** ^1^Cyclotron Research Centre, University of Liège, 8 Allée du 6 Août, Bâtiment B30, 4000 Liège, Belgium; ^2^Walloon Excellence in Life Sciences and Biotechnology (WELBIO), Belgium; ^3^Department of Electrical Engineering and Computer Science, University of Liège, 10 Allée de la Découverte, Bâtiment B28, 4000 Liège, Belgium

## Abstract

Functional and effective connectivity of cortical areas are essential for normal brain function under different behavioral states. Appropriate cortical activity during sleep and wakefulness is ensured by the balanced activity of excitatory and inhibitory circuits. Ultimately, fast, millisecond cortical rhythmic oscillations shape cortical function in time and space. On a much longer time scale, brain function also depends on prior sleep-wake history and circadian processes. However, much remains to be established on how the brain operates at the neuronal level in humans during sleep and wakefulness. A key limitation of human neuroscience is the difficulty in isolating neuronal excitation/inhibition drive in vivo. Therefore, computational models are noninvasive approaches of choice to indirectly access hidden neuronal states. In this review, we present a physiologically driven in silico approach, Dynamic Causal Modelling (DCM), as a means to comprehend brain function under different experimental paradigms. Importantly, DCM has allowed for the understanding of how brain dynamics underscore brain plasticity, cognition, and different states of consciousness. In a broader perspective, noninvasive computational approaches, such as DCM, may help to puzzle out the spatial and temporal dynamics of human brain function at different behavioural states.

## 1. The “Fluid Boundaries” of the Brain: Sleep and Wake

Throughout a 24-hour day, our brain experiences different states. We are awake during the day and we sleep during the night. We generally feel alert in the morning but drowsier in the evening. Thus, the way we feel, react, think, or perform varies over time and depends on the state of our brain. Behavioral states, global cognitive performance, and alertness can be indexed through speed, memory, or vigilance tasks [1]. Although they can provide us with valuable information, they do not give any details about brain activity, which is responsible for how we perform.

The development of neuroimaging technologies has been essential to make huge improvements in our understanding of the human brain. Functional magnetic resonance imaging (fMRI) and positron emission tomography (PET) are widely used to determine which areas of the brain are active during a specific process or task [2, 3]. The obtained images have a high spatial resolution but a low temporal resolution. The information they provide is an indirect measure of neuronal activity. Another family of neuroimaging modalities includes electrophysiological recordings, namely, electroencephalography (EEG), magnetoencephalography (MEG), and local field potential (LFP). These techniques provide direct measures of cortical activity and offer very high temporal resolution but rather low spatial accuracy [4, 5].

Global brain activity fluctuates considerably across the sleep-wake cycle. During wakefulness, the EEG displays fast, low voltage and desynchronized activity. In contrast, during sleep, cortical activity is characterized by slower, higher voltage and more synchronized waves [6]. Cortical activity varies also with time spent awake. For example, sleep deprivation is associated with a global increase in theta (4–8 Hz) activity [1, 7, 8] and beta (13–20 Hz) waves [1, 7]. In a similar vein, cognitive brain function also fluctuates over time. Under a challenging sleep deprivation paradigm [9], fMRI-derived brain areas associated with a working memory task were recruited selectively as a function of time.

These macroscopic observations can be explained by the interaction of two putative processes: sleep homeostasis and circadian rhythmicity. Sleep homeostasis follows the same principles as other homeostatic processes, as originally conceptualized by Bernard in 1865 [10]. It is characterized by an increase or decrease of sleep pressure as wakefulness extends or sleep progresses, respectively. Sleep pressure accumulates or dissipates in a saturating exponential fashion and is almost exclusively dependent on sleep-wake behavior [11]. The second process, the circadian rhythmicity, is an endogenous cycle, whose period is approximately 24 hours. It provides synchrony between an organism's internal biological timing and the external passage of day and night. This process is controlled by the suprachiasmatic nucleus (SCN), which is located in the anterior part of the hypothalamus [12]. In humans, the circadian clock increasingly promotes wakefulness during the day, opposing the gradual buildup in homeostatic sleep pressure [13]. Therefore, this endogenous process is what enables us to have a sleep-wake balance of nearly 8 h–16 h. Beyond normal sleep time, the circadian signal switches to a sleep-promoting signal, and the organism can no longer fight off sleep as efficiently as it does during the day [13]. Throughout sleep, the sleep-promoting signal increases to counter the reduced sleep need. Hence, brain function may heavily rely on sleep-wake history and circadian processes [14].

Studying the brain is not limited to identifying* where* and* when* cortical activity arises. In the last twenty years, much attention has been given to the context-dependent interactions among spatially segregated regions. Neuroscientists now also address the question of* how* distinct areas communicate with each other. Three types of brain connectivity can be defined to account for the interregion interactions [15]. (i) Structural connectivity is defined as the anatomical layout of axons and synaptic connections among neurons. (ii) Functional connectivity refers to the statistical dependency among brain regions. (iii) Effective connectivity designates the direct influence that one region exerts on one another. Both functional and effective connectivity are plastic processes; that is, the communication between distinct cortical areas varies over time.

Functional connectivity in young adults during rested wakefulness and after a night of sleep deprivation was investigated using fMRI [16]. Cortical networks, which are functionally connected under well-rested conditions, became less correlated after prolonged wakefulness, suggesting that highly integrated networks become less integrated during sleep deprivation. In addition, stronger anticorrelation among segregated networks was observed in the first hours of wakefulness than during sleep deprivation. Thus, highly segregated networks become less segregated in sleep deprived conditions. In a similar paradigm, sleep deprivation mainly affected functional connectivity in prefrontal areas [17]. Although these studies are able to* identify* changes in cerebral communication, they do not* explain* which source modifies its information flow towards one another. Functional connectivity estimates are statistical dependencies among cortical regions but they lack a causal description.

Effective connectivity adds this causality component. Experiments focusing on effective connectivity provide insights into which brain areas influence others [25]. To investigate effective connectivity, one often has to define a primary source which is thought to affect others. One way to proceed is to stimulate a specific cortical region by using, for example, transcranial magnetic stimulation (TMS). TMS is a noninvasive method based on Faraday's law of electromagnetic induction [26]. A brief but strong magnetic pulse (1 ms, 1-2 T) is delivered by a coil placed onto the scalp. The rapid change in magnetic field strength induces currents in the brain tissues (i.e., eddy currents) that cause the underlying neurons to be depolarized. The advantage of TMS over sensory stimulation is that it bypasses sensory pathways and subcortical structures to directly reach a desired target [27, 28]. TMS-evoked responses propagated to connected brain areas were investigated under sleep and wakefulness, thus providing an indirect measure of effective connectivity in humans [29]. The rostral portion of the right premotor cortex was stimulated and simultaneous EEG signals were recorded during rested wakefulness, stage 1 of sleep, and nonrapid eye movement (NREM) sleep. TMS elicited a time-locked response that dramatically changed from wakefulness to sleep. During wakefulness, an initial high frequency (20 to 35 Hz) response at the stimulation target was followed by a sequence of lower frequency (8 to 12 Hz) waves until 300 ms. Simultaneously, these waves propagated to connected cortical areas. One could compare this to a stone thrown in a water milieu, creating multiple ripples spreading around the epicentre. When the participants entered stage 1 of sleep, their EEG recordings showed a TMS-evoked response which increased in amplitude. However, subsequent responses were considerably dampened and disappeared after 150 to 200 ms. As soon as they “switched” to NREM sleep, the initial brain response to TMS doubled in amplitude and lasted longer (ca. 150 ms). However, cortical activity at the target returned to its baseline, without displaying any further waves. Furthermore, the response did not propagate beyond the stimulation zone, as if the water milieu had turned to honey, hampering the propagation of ripples.

Altogether, these macroscopic observations suggest that the state of cortical circuits undergoes modifications during the sleep-wake cycle. However, changes in power spectrum, cognitive performance, vigilance, and connectivity are only the “tip of the iceberg.” They necessarily originate from underlying events at the mesoscopic level. The next section provides further insights into how the underlying neuronal dynamics evolve over time.

## 2. Neuronal Dynamics Shape Cortical Behavior

Until recently, sleep (low frequency, high amplitude waves) and wake (high frequency, low amplitude oscillations) were considered as two distinct states. However, there is growing evidence that sleep and wake are not completely dissociated. For instance, Crochet and Petersen recorded low frequency (3–5 Hz) signals in awake immobile mice [30]. As soon as the mice moved, faster waves reappeared, suggesting that oscillatory EEG, which reflects underneath neuronal dynamics, depends on behavioral state but also on whether one is active or not [31]. Slow waves are associated with a large number of hyperpolarized down-states whereas fast EEG oscillations are related to more depolarized up-states [32]. The occurrence of up- and down-states is highly correlated with synaptic excitation and inhibition [33]. Excitation and inhibition are processes occurring concomitantly [34]. An increase in excitation is always followed by a rise in inhibition and vice versa [35]. This observation fostered the concept of “excitation/inhibition balance” [36]. The physiological reason for this equilibrium is to prevent the firing rate of neurons from saturating. It does not mean, however, that excitation and inhibition cancel each other out. Their exact ratio is highly dynamic [35, 37]. Excitation is mostly mediated by glutamatergic cells through short- and long-range projections. On the other hand, inhibition is predominantly controlled by GABAergic (GABA_A_: gamma-aminobutyric acid-A) interneurons, which act mostly locally [35]. Glutamatergic and GABAergic cells have reciprocal interactions. The former excites the latter and vice versa. In essence, excitation and inhibition act concomitantly to generate an action potential, such that a neuron receives massive excitatory signals from glutamatergic cells until it is depolarized [35]. At that point, inhibition inverses the membrane potential curve and the potential drops to a lower value than the equilibrium voltage (hyperpolarization). The neuron then receives some further excitatory inputs to leave the hyperpolarized state. During wakefulness, the number of up-states outnumbers the number of down-states, meaning that the excitation/inhibition balance is in favor of excitation [38]. This balance is reversed during sleep [35].

In rats, cortical neurons fire at a higher frequency after prolonged wakefulness [6], corroborating a previous study [39], which highlighted an increase of excitatory glutamate in the extracellular medium. Specifically, neuronal firing rate increased during the first three hours of sleep deprivation, before it reached a plateau. The appearance of this plateau may coincide with the higher level of GABAergic inhibition also observed in sleep deprived rats [40]. During the recovery sleep following extended wakefulness, average firing rate and synchronous firing decreased [6]. In addition, silencing OFF periods were longer in early than in late sleep while ON periods became longer. OFF periods recorded by LFPs were associated with slow-wave activity (SWA) displayed by the EEG.

In a subsequent study [41], neuronal dynamics were probed in sleep deprived rats and some neurons could briefly enter an OFF period as in sleep. Interestingly, these silencing periods could occur simultaneously in distinct regions or in one cortical area only, but most OFF periods occurred locally. The instances of global and local OFF periods both increased over the course of sleep deprivation. However, the number of global silencing periods increased faster, thus providing some evidence that as sleep pressure builds up, neuronal activity across different cortical areas becomes more synchronized, similar to a sleep state. Moreover, they found that, within a cortical area, some neurons could enter in a “sleeplike” mode while the others maintained or even intensified their activity. In the following NREM sleep, both global and local OFF periods decreased, with the former dropping faster than the latter.

Similar local phenomena have been detected in humans, primarily during sleep. Slow waves and sleep spindles are mainly confined to local regions, particularly in late sleep [42]. Furthermore, local activations of the motor cortex have been observed, such that the activation patterns were characterized by an interruption of SWA and appearance of fast alpha-beta activity [43]. At the same time, other regions showed features of deep sleep. During extended wake states, local increase in theta activity, a marker of sleep need, occurred in task-related regions [44].

Prolonged wakefulness exhibits some characteristics also observed during sleep (i.e., local silencing periods, breakdown in connectivity) [41, 45]. In that sense, extended waking might be temptingly seen as an intermediate stage between the alert wake state and sleep. Similarly, sleep deprivation induces an increase in cortical excitability, which may contrast this view [22]. The increase in glutamate is counterbalanced by an increase in GABA neurotransmitter levels, as a means to maintain an optimal balance between excitation and inhibition [46]. All in all, these observations highlight the complexity of the brain. The mechanisms generating the responses described hitherto are not yet entirely known, but some theories exist. The next section presents some of them.

## 3. Temporal Orchestration of Neuronal Drive

Sleep is widely considered as playing an essential role in synaptic plasticity. The precise mechanisms are still unknown, but some tracks are being proposed. The concepts described here are the “synaptic homeostasis hypothesis” [18, 47], cell-autonomous synaptic scaling [19, 48], and the circadian influence [20].

During the day, organisms interact with their environment. The brain is thus intensively exploited to organize actions, remember events, and pay attention to what is around, and so forth. The intense use of the brain causes synaptic strengthening during the day. The consequences of this synaptic strengthening are, inter alia, higher energy consumption, a greater demand for the delivery of cellular supplies to synapses, and changes in support cells such as glia [18, 47]. Because the organism's metabolism is tightly controlled by homeostasis, synapses cannot continuously be strengthened. Hence, synapses needs to be renormalized at some time. The synaptic homeostasis hypothesis (SHY) suggests that sleep is “the price to pay for plasticity” [18].

**Figure 1:**
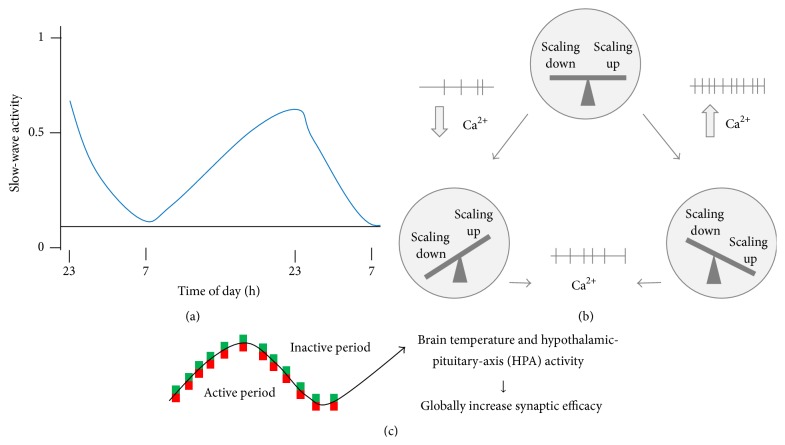
(a) Nonrapid eye movement (NREM) sleep slow-wave activity (SWA) increases with time spent awake and decreases during sleep modified from [18]. (b) Calcium-dependent pathways regulate both scaling up and scaling down, to maintain a balance between excitation and inhibition modified from [19]. (c) Circadian modulation of synaptic plasticity modified from [20].

Slow waves are universal hallmarks of sleep need (Figure 1(a)). SHY proposes that SWA is a sensor* and* a contributor of homeostatic sleep. Slow waves are often associated with burst firing, which may lead to a long-lasting depression of excitatory postsynaptic potentials (EPSPs). Slow waves also causes a cascade of further changes: net synaptic depression, progressive weakening of synapses, reduction in synchrony and firing rates, and finally a decline in SWA. This negative loop proceeds until reaching an equilibrium point where sufficiently low synaptic strength is reached [18].

**Figure 2:**
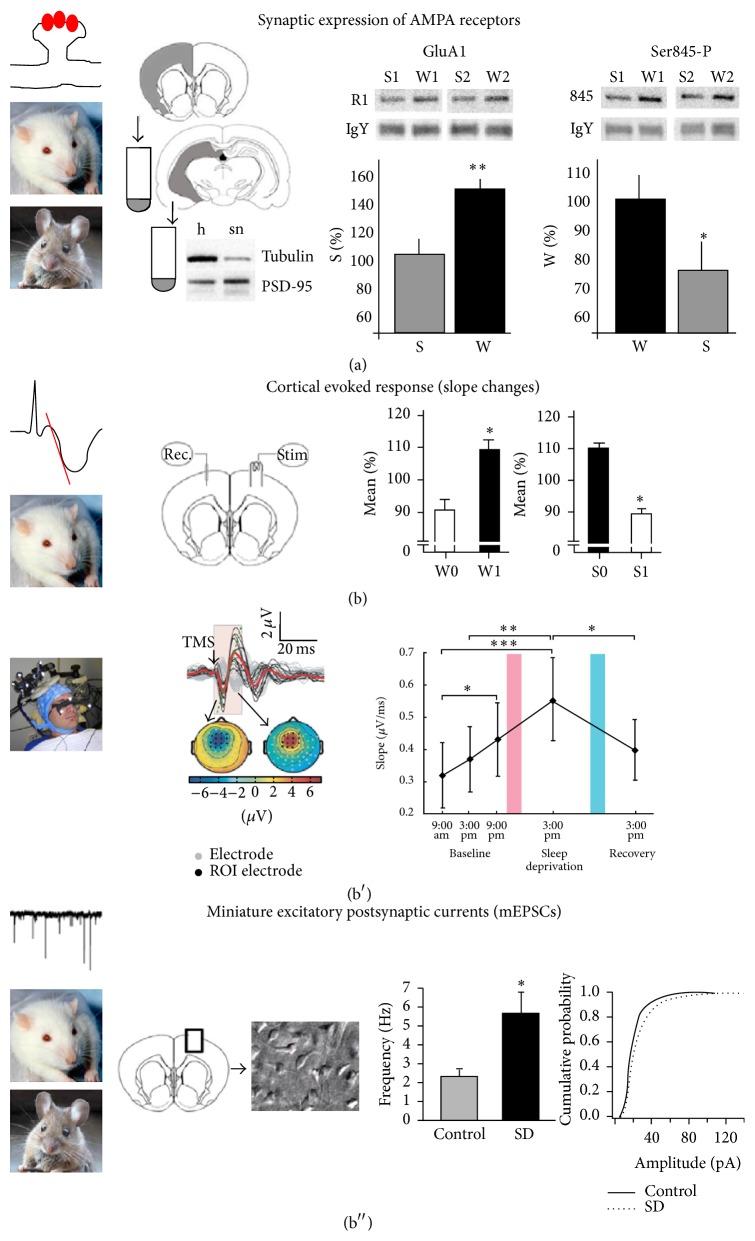
Evidence for synaptic potentiation in sleep deprivation [18]. (a) Experiments in rats and mice show that the number and phosphorylation levels of GluA1-AMPARs increase after wake [18, 21]. ((b), (b′), and (b′′)) Electrophysiological analyses of cortical evoked responses using electrical stimulation in rats [18, 21] and TMS in humans [18, 22] show increased slope after wake and decreased slope after sleep. In (b), W0 and W1 indicate onset and end of ca. 4 hours of wake; S0 and S1 indicate onset and end of ca. 4 hours of sleep, including at least 2 hours of NREM sleep. In (b′), pink and blue bars indicate a night of sleep deprivation and a night of recovery sleep, respectively. (b′′) In vitro analysis of miniature excitatory postsynaptic currents (mEPSCs) in rats and mice shows increased frequency and amplitude of mEPSCs after wake and sleep deprivation (SD) relative to sleep (control) [18, 23].

During wakefulness, the levels of glutamate A1 (GluA1) containing *α*-amino-3-hydroxy-5-methyl-4-isoxazolepropionic acid (AMPA) receptors increase [21]. In drosophila, protein levels of pre- and postsynaptic components, but also the number and size of synapses, are higher during wakefulness [49]. In addition, spine density increases during waking [50]. These changes, among others, participate in what is called synaptic strengthening or long-term potentiation. These phenomena can be indexed through the magnitude of theta power in wake, or NREM SWA in the subsequent sleep [51]. If wakefulness is further extended, such as under sleep deprivation, these wake-related synaptic events may be magnified, as indexed by changes in numerous markers of neuronal excitation described in animals [6, 23, 39, 52] and in humans [22, 53]. In Figure 2, we describe some of the key findings over the past years on how synaptic excitability increases under extended wakefulness.

Why does excitation increase when we extend wakefulness beyond a normal day? When awake, the brain performs multiple tasks demanding energy and cellular supplies [18, 47]. As long as we stay awake, the brain increases its energy consumption and cellular supplies to create new vesicles, for instance. However, this cannot last forever. Imagine a fragile car. During the first kilometres, the ride is safe. Nevertheless, the car starts to get shaky after a while, and the more we drive, the more it shakes. In this setting, something must change to prevent the car from a breakdown. Inhibition is what may prevent “intoxication” of neurons caused by an overload in excitation. Inhibition shapes neuronal spike activity and action potentials in conjunction with excitation to ensure optimal neuronal activity in time and space [35].

The concomitant changes in excitation and inhibition to reach a stable state are one of the key assumptions of homeostatic synaptic plasticity view [19]. Accordingly, there is strong evidence that synaptic scaling is cell-autonomous [19, 54]. Indeed, in vitro experiments where firing rate was blocked led to a scaling up of synaptic strength, while blocking postsynaptic transmission did not have any impact. The scaling up and scaling down of synaptic strength are regulated by calcium-dependent pathways that control the density of AMPA receptors, resulting in a balance between excitation and inhibition [19] (Figure 1(b)). Although homeostasis is probably involved in synaptic plasticity, it may not be the only mechanism and biological clocks may also have a role [20, 55, 56]. For example, circadian modulation over time has been observed in vertebrate ribbon synapses (RSs) [57]. In pineal RSs, their number and size are higher at night than during the day, independently of the animal's endogenous wake propensity (i.e., diurnal or nocturnal) [58]. In retinal RSs, the reverse pattern has been observed [58]. In drosophila, the motor neuron MN5 has more synaptic buttons in the night than in the day, but their size is larger in the day [59]. Furthermore, temperature may explain changes in synaptic strength and morphology, as reported in vertebrate and invertebrate species [20] (Figure 1(c)). A similar scenario may occur in humans. Cortical inhibition in humans, as measured by the duration of cortical silent periods, is higher in the morning than in the evening, irrespective of being measured after a night of sleep or sleep deprivation [60]. The findings indicate that cortical inhibition is linked to time-of-day modulation, which hints at a putative circadian role.

The mechanisms controlling excitation and inhibition are not fully grasped yet. Disentangling the dual roles of sleep homeostasis and circadian clocks in synaptic strength renormalization is intricate. Broadly speaking, our understanding of neuronal mechanisms still needs to be refined. Further studies in animals will allow us to unveil further pieces of this huge puzzle. In humans, however, the portrait is still very blurred. With the exception of pathological cases, such as intracranial EEG recordings in epileptic patients [43], research in humans is solely based on noninvasive techniques that provide indirect measurements about the activity of brain cells [61]. Bridging the gap between microscopic cellular activity and how it is reflected in noninvasive observations is a challenging but essential task. In the next section, we address how in silico computational approaches may offer powerful insights into what is currently unknown on neuronal dynamics.

## 4. From In Vivo to In Silico Activity

Due to the inherent technical and ethical constraints limiting human brain research, further advances rest upon the development of computational models. The idea behind these is to predict the dynamics of group of neurons to better understand macroscopic activity measured by noninvasive imaging modalities. The challenge is twofold. These neuronal dynamics must be appropriately estimated by means of some mathematical techniques, while they have to be properly reflected into neuroimaging data [62].

The behavior of any network is governed by the connections between its distinct nodes. Therefore, to describe neuronal dynamics in a meaningful way, the connections within and between cortical regions must be known. Recently, much interest has been given to building detailed descriptions of the brain network, known as structural connectomes. The human connectome [63–65] is a project which aims to provide a detailed mapping of the brain's connectivity. The term* connectome* is derived from the word* genome*, for which there was also a sequencing project achieved in 2003 [66]. The human brain consists of approximately 10^10^ neurons connected by 10^14^ synapses. In comparison, the human genome comprises around 3.3 × 10^9^ base-pairs, demonstrating the huge challenge to achieve the connectome project. Structural connectomes studies are usually carried out using diffusion-weighted MR imaging (DWI). DWI with whole brain probabilistic tractography investigates structural connectivity in individuals with different alleles of the brain-derived neurotrophic factor (BDNF) gene [67]. BDNF is heavily involved in long-term potentiation and synaptic plasticity [68]. Moreover, it is also implicated in axonal pruning and maintenance, as it prompts the elimination of synaptically silent axonal terminal arbors [69]. Structural connectivity in the forebrain, as well as interhemispheric connectivity, may increase in individuals carrying the* Met* allele in the BDNF gene [67]. Therefore, changes in white matter architecture, as inferred in the structural brain connectivity study abovementioned, might be differentially shaped by BDNF genotype. Another study by Tymofiyeva et al. [70], based on diffusion tensor imaging (DTI) (i.e., an extension of DWI), showed that brain networks become more integrated and less segregated with age.

Besides building structural connectomes, a parallel challenge is to establish a functional brain network and to derive a structure-function mapping [71], which can be derived from MRI or electromagnetic data. Recently, the dynamical correlation between DWI-derived structural connectivity and fMRI BOLD signals was analysed using a sliding window approach [72]. In M/EEG studies, functional connectomes rest upon the analysis of the statistical dependencies of time series using independent component analysis (ICA) [73] or Granger causal modelling (GCM) [74]. ICA is a computational method used to separate a mixture of signals into independent subcomponents [75]. The correlation of independent components is then evaluated to obtain a measure of the functional connectivity between source reconstruction-based cortical regions. On the other hand, the idea behind GCM is that causes precede effects in time. GCM is based on linear vector autoregressive (VAR) models, where the recorded signals can be explained by their own past as well as the past of other signals [74]. The advantage of GCM over ICA is that the former offers a causal description of time series, whereas with ICA, the connectivity measures are purely statistical. However, GCM only regards recorded signals to define functional connectivity but there is no neurobiological explanation of the connectivity [76]. Hence, it is a phenomenological model, as it forgoes any biological attempt to explain why variables interact the way they do and simply describes their relationship based on observations [76, 77].

To cope with this limitation, functional connectomes can also be built from physiological models, which are known as dynome [78]. Dynome focuses on brain rhythms in local structures, without explicitly accounting for the activity at the single-neuron level or individual axons joining cortical areas. Dynome operates on a larger scale, the mesoscale (e.g., a cortical column), as the human brain appears to be built from large populations of neurons performing the same function [62]. They are based on detailed biophysical mechanisms, which are extremely complex as different rhythms can appear in a single cortical region (i.e., cross-frequency coupling) and several neuromodulators can have different effects on frequency bands [78]. The dynome models for different cortical regions and different frequency ranges can then be assembled as building blocks to explain behavior at a macroscopic scale [78]. For example, gamma oscillations in rodents are highly correlated to perisomatic inhibition and are modulated by slower rhythms [79], including modulation by theta waves [80]. In essence, this cross-frequency coupling of rhythms generates a multiscale timing mechanism [31] between different states of vigilance. Ultimately, it allows for an effective mechanism linking cortical circuits.

A complement approach to dynome is Dynamic Causal Modelling (DCM). Despite the similarities, it is not a dynome as such, because it relies on more abstract biophysical models that offer an approximation of the hidden neuronal states. Briefly, DCM is an approach to investigate how effective connectivity is affected by context, based on a biologically realistic generative model [81]. DCM has been extensively used in animal and human models, ranging from local field potentials (LFP) intracranial recordings to noninvasive approaches, such as hemodynamic or EEG responses. DCM allows for the comprehension of how brain dynamics underscore cognition [82] and different states of consciousness [83]. The next part explains in detail the principles underlying DCM.

## 5. Dynamic Causal Modelling

Dynamic Causal Modelling (DCM) is a computational approach that allows one to quantify the effective connectivity between and within brain areas and to investigate how the parameters of effective connectivity are influenced by experimental factors. In other words, DCM probes how a given experimental manipulation affects the strength of cortical connections [82–84]. DCM was introduced by Friston et al. [2] and was first intended for fMRI data but was later extended to model electromagnetic time series. The idea is to build a network of nodes (i.e., cortical areas) that interact through extrinsic connections. Each cortical area of the network is represented by a biologically plausible neuronal model [24]. Based on current physiological knowledge about connectivity parameters, this* forward model* generates realistic electromagnetic data [81]. The recorded and predicted data are then compared and the parameters of the neuronal model are adjusted to improve data prediction. This step is called the* inverse problem* [85]. Overall, DCM proceeds in a loop between the forward and inverse problems until all the parameters of the forward model have reached values providing an optimal prediction of the data.

The next parts of this section focus on DCM for electromagnetic signals. First, we provide an in-depth description of the neuronal model. Next, we explain how the forward and inverse procedures are used to obtain the values portraying effective connectivity. Finally, we catalogue the different types of DCM analyses and present some DCM applications.

### 5.1. The Neuronal Model

**Figure 3:**
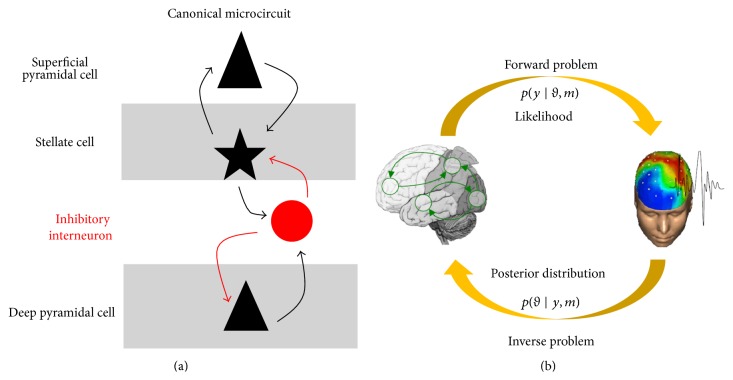
(a) Canonical microcircuit (CMC) epitomizes a given cortical column and comprises four neuronal populations connected through excitatory and inhibitory projections (modified from [24]). (b) Schematic diagram of the DCM algorithm functioning. The forward problem rests upon a neuronal model (as for (a)) and provides a likelihood function, which is then used in the inverse problem to derive a posterior distribution for the neuronal parameters.

The neuronal model is used to emulate the activity of regions of interest. Each cortical region is identically modelled with a number of interconnected neuronal populations, as originally described by Jansen and Rit [86]. This model comprises three neuronal populations, albeit the most recent neuronal models contain four populations: (i) excitatory spiny stellate cells in granular layer IV, (ii) inhibitory interneurons in supragranular layers, (iii) superficial pyramidal cells in supragranular layers, and (iv) deep pyramidal cells in infragranular layers [87] (Figure 3(a)). The four neuronal populations of each cortical column are interconnected through intrinsic connections in agreement with observations in animals [87]. The neuronal model can explain cortical activity in different ways. The neuronal dynamics can be described by the mesoscopic properties of the populations (convolution-based models) [81] or they can encompass single-cell electrophysiological properties (conductance-based models) [88]. This latter class of neuronal model takes into account key ionotropic receptors, such as AMPA, GABA_A_, and NMDA (N-methyl-D-aspartate). Different brain regions are linked by excitatory extrinsic connections. Anatomically, intrinsic connections link neuronal population within the same gray matter region, while extrinsic connections cross white matter to join distinct cortical areas. A further distinction between intrinsic and extrinsic connections lies in the delay of their effect, with intrinsic connections having smaller delays (≈2 ms) than long-range extrinsic connections (≈16 ms) [89]. Following the concept of hierarchical organization of the cortex formulated by Felleman and Van Essen [90], extrinsic connections are directed and can be classified as (i) forward, (ii) backward, or (iii) lateral. Forward or bottom-up connections originate in agranular layers (i.e., supragranular and infragranular layers) and terminate in layer IV. Backward or top-down connections join agranular layers. Lateral connections originate in agranular layers and target all layers.

### 5.2. The Forward and Inverse Problem

Based on physiological assumptions (i.e.,* priors*) about synaptic parameters, the neuronal model provides an estimation of the direct cortical activity. However, the electromagnetic time series one has at hand is only a reflection of this activity. The observation model provides this conversion by accounting for the propagation of signals through head tissues [89]. Altogether, the neuronal and observation models render the forward model and yield a* likelihood* function, which characterizes how well the predicted data approximate the recorded data [91]. Thus, the forward problem is a generative model to synthetize data. In essence, it can be seen as the prediction step of DCM. For the forward model to optimally explain the observed signal, the parameters tuning it and the predicted data must be adjusted. During this step, we say that the DCM model is* inverted*. This inversion is performed in a probabilistic framework, known as Bayesian inference [85]. The parameters of the neuronal model are obtained by relying on Bayes' rule:

(1) $$\begin{array}{c} p\left(\;\theta \;|\;y,m\;\right)=\frac{p\left(\theta \;|\;m\right)p\left(y\;|\;\theta ,m\right)}{p\left(y\;|\;m\right)}, \end{array}$$

where *θ*, *y*, and *m* correspond to the model parameters, the observed data, and the model under consideration, respectively. The* posterior* distribution *p*(*θ*∣*y*, *m*) of the parameters is built upon the* likelihood p*(*y*∣*θ*, *m*),* priors p*(*θ*∣*m*), and* model evidence p*(*y*∣*m*) distributions.

Nevertheless, this formula cannot be evaluated explicitly. Instead, it is approximation using an iterative process called variational Bayesian technique [92]. Once the synaptic parameters are adjusted by this update step, a new likelihood function is evaluated in the forward problem, and so forth, until the neuronal parameters reach an optimal solution (Figure 3(b)).

One may formulate different hypotheses concerning how cortical areas are connected. For example, two regions can communicate through a unique unidirectional connection or they can be linked by bidirectional connections. The model evidence quantifies the properties of a good model, by explaining the data as accurately as possible and with minimal complexity [15]. Thereof, different hypotheses, that is, connectivity architectures, can be compared, using Bayesian model selection (BMS) [93], which selects the most likely model. A palpable example is the perturbation of neuronal dynamics by sensory input among cortical circuits [5]. This information may be relayed through top-down and/or bottom-up connections across frontotemporal regions (i.e., primary auditory cortex, superior temporal gyrus, and inferior temporal gyrus). By applying BMS, it was possible to identify the importance of top-down connections in the predictive coding of sensory information [5].

### 5.3. DCM Applications

DCM allows for the analysis of different types of data, including noninvasive EEG and MEG time series and intracranial LFP data. Furthermore, both evoked responses and spontaneous activity can be studied with DCM. Currently, four variants of DCM exist to analyse different data or to answer different questions: event-related potentials, steady-state responses, induced responses, and phase-coupling. DCM for event-related potentials (ERP) [94] is designed for data acquired when a known and deterministic input is applied on specific cortical regions. The input is a stimulus that perturbs the neuronal dynamics and elicits an evoked response. The signals are considered in the temporal domain over typically short time windows (<1000 ms) around the event. The second type of DCM analysis is designed for steady-state responses of the brain; that is, no stimulus is used to trigger a brain response. When one is looking at spontaneous cortical activity, it is more efficient to consider the data in the frequency domain by assessing their power spectrum. This is the approach used in the DCM for cross-spectral densities (CSD) [89]. Chen et al. [95] introduced an “induced response” variant of DCM. It models time-varying frequency power as the response to a stimulus or a task. Contrary to the aforementioned DCM flavours, this type of DCM does not rely on a neurophysiological motivated model, but rather on a phenomenological model, where the neuronal parameters are hidden and not explicitly inferred. Finally, Penny et al. [96] provided another type of DCM based on the analysis of phase-coupling, where the neuronal dynamics are expressed in terms of neuronal synchronisation processes using weakly coupled oscillators. Similar to DCM for induced response, DCM for phase-coupling rests upon a phenomenological model. Eventually, it infers if different cortical sources are synchronised or not.

Hereafter, we illustrate how DCM may offer putative insights into hidden neuronal dynamics across different paradigms.

An unequivocal aspect of global cortical functioning is the adaptation to changes in the environment, an ability coined as “brain plasticity” [97]. A myriad of factors may trigger cortical plastic changes. Among them is learning, which, broadly speaking, may be associated with repetition-dependent plasticity in the brain. These learning-induced changes may impact on intrinsic and extrinsic connections within a cortical network. By applying a DCM for ERP associated with a given learning paradigm, one may infer how connectivity parameters evolve over time. Brain connectivity has been shown to change as participants heard a repeated acoustic stimuli (roving mismatch paradigm) [84]. Based on EEG recordings and by applying DCM for ERP, the connectivity strength in a network involving the primary auditory cortex and superior temporal gyrus decreased with repetition. Plasticity may thus be a key mechanism for adaptation to a specific stimulus across distributed brain systems [98].

An important question in human neuroscience is how discrete synaptic events may underpin different states of consciousness and behavioral states. The former was tested in a DCM study, whereby the effective connectivity between the frontal and parietal cortices was investigated in severely brain-damaged patients [83]. Intriguingly, the transmission of information flow (top-down connectivity) in the frontoparietal cortex differed between patients in vegetative state and controls. The frontoparietal cortex is involved in the explicit processing (awareness) of stimuli [99]. Therefore, “impairments” in the effective connectivity in this brain circuitry might be critical for the perception of consciousness.

As mentioned earlier, DCM has been applied in “mice to men” studies, as a means to infer neuronal states in different experimental settings. One key approach when using DCM is the capacity to infer how specific neuronal parameters encode for changes in EEG spectral oscillations [82]. DCM for CSD is the “DCM flavour” for such premise and is intended for spontaneous activity [100] and/or pharmacological experiments [82].

A previous in vivo study has shown that ketamine reduces theta activity and increases gamma activity in the hippocampus in mice [101]. With that assumption in mind, neuronal mechanisms underpinning the variations in frequencies in the hippocampus and the prefrontal cortex were investigated in ketamine-doped rats [102]. Using DCM for CSD, a reduction in top-down connectivity from the medial prefrontal cortex to the dorsal hippocampus was observed under ketamine. The excitatory NMDAR-mediated bottom-up connectivity from the prefrontal cortex to the hippocampus also decreased with ketamine doses. In contrast, they noticed an increase in the excitatory AMPAR-mediated bottom-up connectivity. All in all, this study provides some insights into how ketamine induces a breakdown in corticohippocampal connectivity. In humans, NMDAR dysfunction and theta-gamma rhythm abnormalities play a key role in neuropsychiatric disorders, such as schizophrenia [103]. Therefore, the insights provided by this DCM study in rodent models may offer a framework for how aberrant mechanisms of cortical information flow underpin neuropsychiatric ailments.

DCM has also been applied to in vivo assays of ongoing synaptic processing underlying human cognition [82]. Magnetoencephalographic (MEG) measurements were acquired from participants during a working memory task, under a pharmacological (dopaminergic) challenge. The rationale for this approach is the critical role that the dopaminergic system plays in working memory [104]. DCM for CSD was applied to reconstructed signals from the prefrontal cortex. In essence, specific synaptic mechanisms, which included synaptic transmission via excitatory AMPA and NMDA and inhibitory GABA_A_ receptors and glutamatergic inputs to layer IV, may be modulated by L-Dopa [82]. Thus, changes in these ionotropic receptors may be associated with the synaptic effects of dopamine. Importantly, better performance in the working memory task was associated with these changes in DCM-derived ionotropic receptor levels. These results provide a novel framework to noninvasively infer how hidden synaptic events mediate cognitive processes in humans.

DCM for CSD can also be applied onto recordings of spontaneous activity, without any influence of a drug. It has so far been used predominantly on data acquired in pathophysiological conditions such as epilepsy. An epileptic state arises from a sudden increase in excitation triggered by a deviation of the interaction between pyramidal cells and inhibitory interneurons from its normal regime [105]. A recent study showed that epileptogenesis is also characterized by a slow drift in intrinsic connectivity within and surrounding the ictal zone [100]. Using DCM for CSD, increased intrinsic connectivity from inhibitory interneurons to superficial pyramidal cells was detected at seizure onset, followed by a gradual decrease thereafter.

The activity of neocortical neural circuits is powerfully modulated by subcortical inputs [106]. However, recording subcortical activity using noninvasive EEG recordings is a challenge. Spatial EEG sensitivity depends on biological parameters, such as (i) distance between neuronal populations and EEG sensors and (ii) the complex cellular architecture of deeper sources [107]. Thus, deep brain structures are typically considered as electromagnetically “silent” [108]. However, as subcortical activity influences cortical activity through subcortical-cortical connectivity, its changes may be indirectly inferred from EEG signals. If one uses biophysical models of EEG activity, in which synaptic activity is generated by artificial neural networks, then parameters of subcortical activity can be included in a model as modulating cortical sources, which are those mostly detected at the EEG level [108]. Within the DCM framework, both variables can be inferred from actual EEG data [109]. This assumption was highlighted by a study investigating the mechanisms for anesthesia-induced loss of consciousness [110]. By applying DCM for CSD to data from wakefulness, mild sedation, and loss of consciousness, spectral changes across all behavioral states involved changes in corticothalamic interactions. Compared with wakefulness, mild sedation was accounted for by an increase in thalamic excitability that did not further increase during loss of consciousness. Conversely, loss of consciousness was associated with a decrease in backward corticocortical connectivity from frontal to parietal cortices, while thalamocortical connectivity was unaffected. Furthermore, DCM for CSD data in Parkinson patients inferred that connections to and from the subthalamic nucleus were strengthened and promoted beta synchrony, in untreated relative to treated Parkinson state [111]. These results* per se* emphasize the role of subcortical modulation in cortical dynamics.

Strikingly, much still remains to be known about the hidden neuronal events underpinning different sates of vigilance. To date, our understanding of putative cortical mechanisms accounting for sleep and wakefulness is primarily derived from in vivo studies in animal models (for a review, see [112]) and data from epileptic patients (for a review, see [113]). Spontaneous activity in sleep and wake may be investigated using DCM for CSD as in the previous studies.

Despite the potential to unveil hidden neural states, DCM has drawbacks. As it relies on abstract, neural mass models [24, 114], it may not accurately reflect biophysical properties critical to some neural computations. Chief among these is absence of neuromodulatory factors, including cholinergic, serotoninergic, and dopaminergic activity, that impact on cortical dynamics [61]. Another critical aspect is that DCM cellular parameters do not represent single-cell properties, but rather those of a cortical macrocolumnar neuronal network. Thus, it is designed to unravel functional dynamic properties of a neuronal ensemble. In itself, this hinders the understanding of single-cell activity, currently inferred only from direct intracranial recordings in epileptic patients [113]. Indeed, a key limitation in human neuroscience is the understanding of how brain circuits operate in fine spatial-temporal dynamics in vivo. Thus, computational approaches become an attractive choice at hand to explore these latent neuronal dynamics. In this scenario, DCM may offer a unique insight into these dynamics by exploring the effective connectivity within and between cortical regions.

In light of the studies illustrated in this section, we can ask whether DCM is indeed a dynome. Dynome and DCM address the same questions, namely, how brain activity is orchestrated among regions and how it varies depending on the context. In Kopell et al. [78], it was argued that DCM relies on abstract biological generative models. Although this was true for the earliest models, the latest ones account for subtle features like the presence of four different neuronal populations, the introduction of electrophysiological properties of neurons [88], and the dependence on space [115]. These ingredients make the latest generative models biologically meaningful. To foster even more their reliability, DCM neuronal models would have to include the role of glial cells, internal (e.g., thermal) noise in neurons, and dendritic backpropagation [15]. Regarding the connections between brain nodes, dynome and DCM share the fact that they do not model the brain as an entity; yet, they are different. In DCM, several sources can be modelled and interact by making connections among them. On the other hand, dynome investigates local networks and treat them as independent building blocks [78], which is a biological deficiency on its own.

In short, both DCM and dynome approximate the dynamics of the brain. In that sense, it is hard to say that one is more physiological than the other. Notwithstanding their differences, DCM, dynome, and other methodological techniques that use varying temporal scales allow us to better apprehend the rich spatiotemporal landscape of brain dynamics.
